# Vertical porous 1D/2D hybrid aerogels with highly matched charge storage performance for aqueous asymmetric supercapacitors

**DOI:** 10.3389/fchem.2025.1550285

**Published:** 2025-02-28

**Authors:** Panji Xu, Kunhua Quan, Xiyuan Wei, Yubing Li, Shuaikai Xu

**Affiliations:** School of Physics Science and Technology, Guangxi University, Nanning, China

**Keywords:** asymmetric supercapacitors, hybrid aerogels, MXene, graphene, optimized ion pathway

## Abstract

Asymmetric supercapacitors (ASCs) have attracted widespread attention because of their high energy density, high power density and long cycle life. Nevertheless, the development of anodes and cathodes with complementary potential windows and synchronous energy storage kinetics represents a pivotal challenge. We propose to construct nanochannel-coupled vertically porous CNF/Ti_3_CNT_x_ and CNF/rGO hybrid aerogel electrodes via a unidirectional bottom-up cryoprocess. The vertically porous structure will greatly shorten the ion diffusion path and enhance the charge/ion transfer/diffusion kinetics, and the inserted cellulose nanofibers (CNFs) will impede the re-stacking of the nanosheets and enlarge the interlayer nano-channels, thus improving the accessibility of electrolyte ions. Ultimately, all-solid-state ASCs assembled based on nanochannel-coupled vertically porous MXene and graphene aerogel can achieve an excellent energy density of 20.8 Wh kg^−1^ at 2.3 kW·kg^−1^, a high multiplicity performance, and retains 95.1% of energy density after 10,000 cycles. This work not only demonstrates the great superiority of nanochannel-coupled vertically porous hybrid aerogels, but also provides an effective strategy for designing asymmetric supercapacitor electrodes with matched structural and electrochemical properties.

## 1 Introduction

Supercapacitors (SCs) have been widely used in portable and wearable electronic devices due to their high power density, long cycle life and rapid charge–discharge rate (Yu et al., 2014; Salanne et al., 2016; Wang et al., 2016; Xiong et al., 2020). However, the limitation of low energy density hinders its practical application. According to the energy density equation E = 1/2CV^2^, the most effective way to increase the energy density of supercapacitors is to develop asymmetric supercapacitors (ASCs) (Yu et al., 2014; Zhang and Pan, 2015; Shao et al., 2018; Simon and Gogotsi, 2020; Huang et al., 2022), which effectively broaden the operating voltage by combining two suitable positive and negative electrode materials, thus increasing the energy density (Yan et al., 2014; Chodankar et al., 2020; Choi et al., 2020). Therefore, the development of matched positive and negative materials with complementary potential windows and well-coordinated charge storage kinetics is particularly important to synergistically optimize the energy and power capabilities of ASCs (Yan et al., 2014; Chodankar et al., 2020; Guo et al., 2022; Xu et al., 2024).

In recent years, two-dimensional (2D) nanomaterials have attracted great research interest in the field of energy storage due to their unique atomic thickness and properties. Graphene and MXenes, as two well-known 2D nanomaterials, have received much attention in the field of asymmetric supercapacitors. Graphene, with its large theoretical specific surface area, excellent electrical conductivity and high potential range, is an ideal candidate for cathodes (Zhu et al., 2010; Zhu et al., 2011). MXenes, as emerging two-dimensional nanomaterials, which have the advantages of both metallic conductivity and surface hydrophilicity. The abundance of functional groups on the surface facilitates rapid surface redox reactions and interfacial ion transport, making MXenes suitable for use as anodes (Anasori et al., 2017). By matching the low electrochemical window of MXenes with the higher electrochemical window of graphene, a broad range of cell voltages can be achieved, and the stable potential range of each can be fully utilized in asymmetric designs to achieve high energy density.

However, strong van der Waals interactions between neighboring nanosheets (Xu et al., 2023) and the inevitable re-stacking or aggregation of pristine graphene and MXene nanosheets (Mendoza-Sánchez and Gogotsi, 2016; Wu et al., 2020; Li et al., 2022) during electrode preparation tends to greatly reduce the utilization of the active substance surface and impede the charge/ion transport during repetitive charging/discharging processes. Some effective strategies to solve the above problems are through the design of heterostructures and morphology control (Du et al., 2023; Liang Y. et al., 2024), such as reduced graphene oxide/manganese dioxide or carbon nanotube (CNT) composite aerogels (Liang X. et al., 2024), loosely disordered MXene/reduced graphene oxide (rGO) 3D skeletal framework electrode (Zhu et al., 2024), cellulose nanofibers (CNF)/MXene and porous electrode design (Zhao et al., 2015; Lukatskaya et al., 2017; Yan et al., 2017; Tian et al., 2019). Among them, compared to the design of two 2D nanomaterial heterostructures, the use of one-dimensional cellulose nanofibers to form nano-supporting channels between the MXene layers fundamentally suppresses the overlap of 2D materials. In addition, CNF, with excellent flexibility and moisture absorption, improves the overall mechanical properties of the electrode structures and enables the materials to have good electrolyte storage capacity. However, in these structures, the diffusion of ions through the electrode thickness is still slow (Ghidiu et al., 2014), and it is time-consuming and difficult to meander through the cumbersome interlayer nano-channels to interact with the entire electrode. Therefore, it is necessary to further optimize the ion transport path, such as aligning the MXene nanosheets vertically, to increase the ion diffusion speed and shorten the ion diffusion path (Du et al., 2023). On the other hand, realizing high electrochemical properties of MXene-based composites with large mass loading and thickness is crucial for practical applications (Xu et al., 2023). To date, excellent rate performance has been reported for porous graphene-based electrodes, hierarchical porous MXene films and MXene aerogels (Yue et al., 2018). In summary, the construction of 3D MXene or graphene-based hybrid aerogels with interlayer nano-channels coupled with vertical ion diffusion pathways is expected to be an effective method to promote the application of 2D materials in high-performance energy storage devices.

In this work, cellulose nanofibers were selected to be combined with rGO and Ti_3_CNT_x_ to construct CNF/rGO and CNF/Ti_3_CNT_x_ hybrid aerogels for positive and negative electrodes of high-performance ASCs, respectively. The vertical ion diffusion paths inside the hybrid aerogels were designed by a unidirectional bottom-up freezing process. The intercalated CNF can effectively resist the re-stacking of nanosheets, expand the interlayer nano-channels, and improve the active surface utilization of the material. In addition, the incorporation of CNF improves the mechanical strength of the hybrid aerogel and forms a good electrolyte storage in the electrode. The nano-channel-coupled vertical porous structure greatly shortens the ion diffusion path and improves the accessibility of the electrolyte ions, thus realizing high-speed charge transfer. The assembled all-solid-state ACS achieves a broadened voltage window, high-speed charge/ion transfer/diffusion kinetics, and a stable electrode structure. It can provide a high energy density of 20.8 W h kg^−1^ at a power density of 2.3 kW kg^−1^ with a wide voltage window of 1.5 V. It also has a very high cycling stability with 95.1% capacitance retention after 10,000 cycles. The optimization of ion transport paths and structural stability in the electrode by this structural design of nanochannel-coupled vertical porous provides an attractive strategy for the design of high-performance electrodes for MXene and other 2D nanomaterials.

## 2 Experimental section

### 2.1 Synthesis of few-layer of Ti_3_CNT_x_ dispersion

Few-layer Ti_3_CNT_x_ nanosheets were synthesized by etching Ti_3_AlCN (CarbonFone Technology) using an acid mixture of LiF (aladdin)/HCl (Sinopharm). Briefly, 4.8 g of LiF was dispersed in 60 mL HCl (12 M), then 3 g of Ti_3_AlCN powders was slowly added into the acid mixture of LiF/HCl (Sun et al., 2020). The mixture was continuously stirred at 30°C for 24 h, and the products were washed and centrifuged several times with deionized water until the supernatant turned black. Finally, few layer Ti_3_CNT_x_ dispersion (2 mg mL^−1^) can be obtained by delaminating the sediment through ultrasonic treatment for 30 min.

### 2.2 Preparation of GO homogeneous solution

The GO (CarbonFone Technology) was prepared through the modified Hummers method using graphite powder. Then 0.5 g of GO was dissolved in 50 mL of water for ultrasonic treatment for 2 h to obtain a homogeneous GO dispersion solution (Xu et al., 2010).

### 2.3 Preparation of CNF dispersion

Cellulose nanofibers (CNF, ScienceK) were prepared by TEMPO oxidation. 10 mL of 10 mg mL^−1^ CNF gel was added to 40 mL of deionized water for ultrasonic dispersion treatment for 2 h. A homogeneous CNF dispersion was obtained with a concentration of about 2 mg mL^−1^.

### 2.4 Preparation of CNF/Ti_3_CNT_x_ composite aerogel

The CNF dispersion was poured into the prepared lesser layer of Ti_3_CNT_x_ dispersion to produce a mixed dispersion and stirred thoroughly. Two or three drops of 3 M sulfuric acid solution were subsequently added to the mixed dispersion to break the electrostatic equilibrium between the nanosheets, which led to the gelation of CNF and MXene nanosheets. The mixed slurry was then dropped onto a copper plate and then rapidly frozen. After further freeze-drying process, CNF/Ti_3_CNT_x_ composite aerogels were produced. As a comparison, CNF/Ti_3_CNT_x_ composite aerogels with different CNF contents (2, 5, 8, 10 wt%) were prepared and labeled as 2% CNF/T11, 5% CNF/T11, 8% CNF/T11, and 10% CNF/T11, respectively.

### 2.5 Preparation of CNF/rGO composite aerogel

The CNF dispersion was homogeneously mixed with a few layers of GO dispersion in which the mass percentage of CNF was 5%. Two or three drops of 3 M sulfuric acid solution were added to the mixed dispersion to break the electrostatic equilibrium between the nanomaterials, which led to gelation of CNF and GO nanosheets. Subsequently the mixed colloid was dropped on a copper plate and then rapidly frozen. After further freeze-drying process, CNF/GO composite aerogels were produced. Finally, the 5% CNF/rGO composite aerogel was successfully obtained by annealing the CNF/GO composite aerogel in an argon environment at 300°C for 2 h.

### 2.6 Assembly of the asymmetric supercapacitors

To ensure the optimal performance of the assembled asymmetric device, it was crucial to maintain charge balance (Q_cathode_ = Q_anode_) between the cathode and the anode. The active mass of the electrodes material at charge balance was calculated using the equation Q_electrode_ = C_electrode_ × m × ΔE, based on the potential window (ΔE) and specific capacitance (C_electrode_) of the cathode and anode in the three-electrode system. From these conclusions and experimental findings, the mass ratio of negative and positive electrodes is 1/1.7. Subsequently, ASC devices in a 2 M H_2_SO_4_ electrolyte were assembled using the CNF/rGO electrode and CNF/Ti_3_CNT_x_ electrode with the Celgard 3,501 separator. The all-solid-state ASC devices were similarly assembled using a PVA-H_2_SO_4_ electrolyte.

### 2.7 Materials characterizations

The thickness of the prepared few-layer Ti_3_CNT_x_ nanosheets was confirmed by atomic force microscope (AFM, Bruker Multimode 8). A powder X-ray diffractometer (XRD, Bruker, D8 ADVANCE, Cu Kα radiation (λ = 0.15406 nm)) was used to analyse the phases of the related nanomaterials in this work. The morphology and structure of the samples were characterized using a scanning electron microscope (SEM, Zeiss Gemini 300). The contents of elements of composite aerogel can be characterized by energy dispersive spectroscopy (EDS). The specific surface area and pore size distribution of the sample were assessed using Tristar II 3020 Version 3.02. The conductivity of the electrodes was measured with an RTS-8 four-probe resistivity meter (China). The contact angle was measured by a dynamic contact angle testing instrument (OCA40, Dataphysics, Germany) equipped with a dynamic image capture camera. Electrochemical measurements. The electrolyte with a volume of 10 μL was placed on the surface of the electrodes and allowed to stabilize for 10 s before a picture was taken.

### 2.8 Electrochemical measurements

The electrochemical performances of the hybrid aerogels were evaluated by an electrochemical workstation (Ivium-n-Stat). Electrochemical tests, such as cyclic voltammetry (CV), galvanostatic charge/discharge (GCD), and electrochemical impedance spectroscopy (EIS), were performed in 2 M H_2_SO_4_ aqueous electrolyte, where the hybrid aerogels were directly used as the working electrodes, activated carbon as the counter electrode, and Hg/HgSO_4_ as the reference electrode. A potential amplitude of 10 mV was applied on the working electrode to record the corresponding EIS in the set frequency range (100 kHz-10 mHz) at open-circuit potential.

The gravimetric capacitance ( $C_{m}$ ) was calculated according to the following Equation 1:

$$C_{m}=\frac{\int idU}{∆Umv}=\frac{S}{2∆Umv}$$ (1)

Where i is the current, ∆U is the potential window, *v* is the scan rate, m is the electrode mass and S is the integral area of the CV curve.

The gravimetric energy density ( $E_{m}$ ) and power density ( $P_{m}$ ) of the ASCs were calculated based on the following Equations 2, 3:

$$E_{m}=\frac{\int Uidt}{m}=\frac{1}{2}C_{m}U^{2}$$ (2)

$$P_{m}=\frac{E_{m}}{∆t}$$ (3)

Where m represents the total weight of electrode materials and ∆t represents the discharge time.

The mass loading of the positive electrode to the negative electrode was calculated based on charge balance according to the following Equation 4:

$$\frac{m_{+}}{m_{-}}=\frac{C_{-}∆E_{-}}{C_{+}∆E_{+}}$$ (4)

Where $m_{+}$ and $m_{-}$ are the mass loading of the cathode and anode, $C_{-}$ and $C_{+}$ are the gravimetric capacitance of the cathode and anode in three-electrode system, and $∆E_{-}$ and $∆E_{+}$ are the potential window of the cathode and anode in three-electrode system.

The $D_{H^{+}}$ can be calculated by utilized the three Equations 5–7 based on EIS:

w=2πf (5)

$$Zre=R+\sigma w^{-0.5}$$ (6)

$$D_{H^{+}}=\frac{0.5R^{2}T^{2}}{A^{2}n^{4}F^{4}C^{2}\sigma^{2}}$$ (7)

The f is test frequent, R (gas constant) is 8.314 J mol^−1^ K^−1^, T (Kelvin temperature) is 293.15 K, A (area of electrodes) is 0.28 cm^2^, F (Faraday constant) is 96,485 C mol^−1^, σ is Warburg coefficient, n is electronic transfer number per molecule, C is molar concentration H^+^.

## 3 Results and discussion

### 3.1 Morphology and composition of prepared materials

**FIGURE 1:**
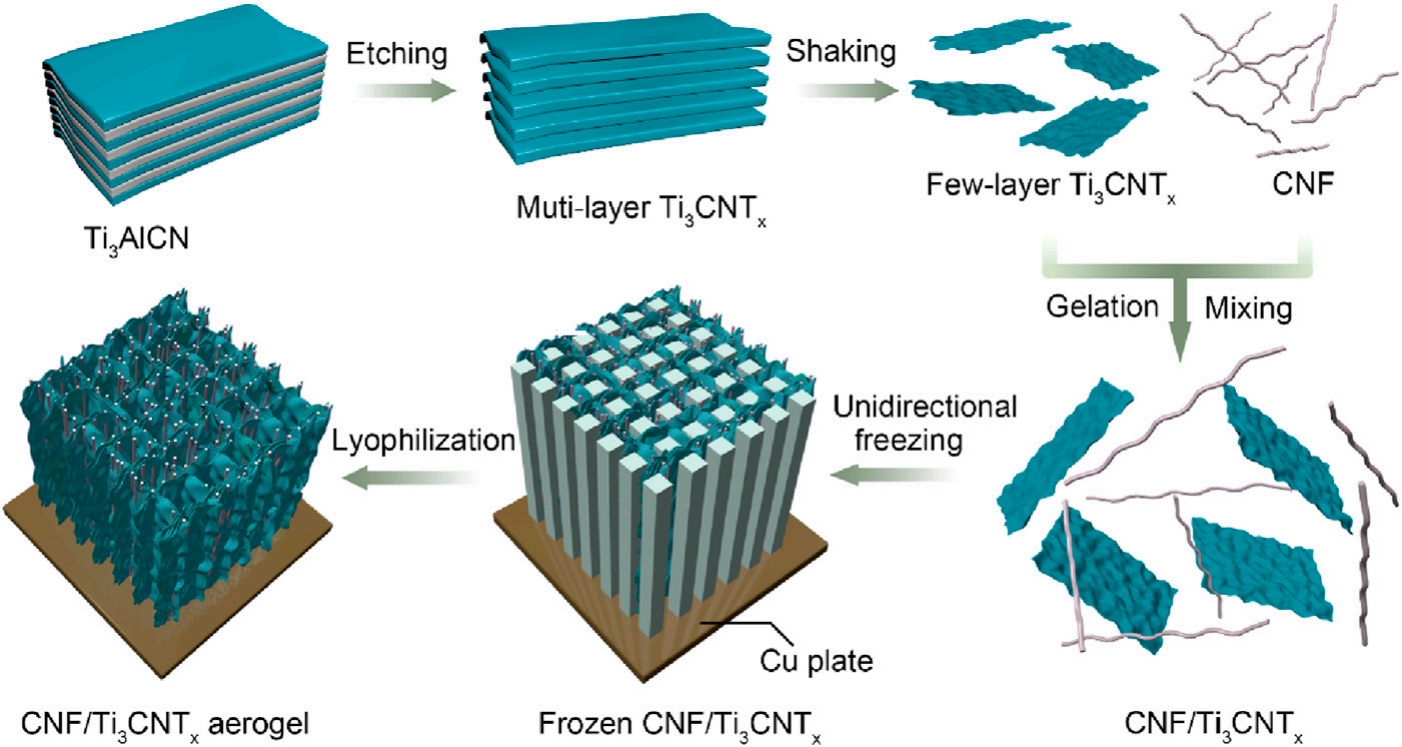
Schematic diagram of preparation process of vertical porous CNF/Ti_3_CNT_x_ hybrid aerogels.

The CNF/Ti_3_CNT_x_ hybrid aerogels were prepared by a bottom-up unidirectional freezing process, which is a low-cost, simple and mass-producible method for the design of three-dimensional structures. The synthesis process of CNF/Ti_3_CNT_x_ hybrid aerogels is schematically shown in Figure 1. Several layers of Ti_3_CNT_x_ nanosheets were prepared by selectively etching the Ti_3_AlCN precursor using HCl/LiF mixed etchant. Cellulose nanofiber (CNF) dispersion was prepared by TEMPO oxidation. The CNF dispersion was poured into the prepared few-layer Ti_3_CNT_x_ dispersion and stirred well to form a mixed dispersion. Then a few drops of acid solution were added to the mixed dispersion to break the electrostatic equilibrium between the nanosheets to gel the CNF and MXene nanosheets. Subsequently, the resulting slurry was uniformly applied to the upper surface of the copper plate and further frozen from bottom up. As the cooled slurry freezes, the CNF and MXene nanosheets begin to aggregate around the growing ice crystals until the slurry is completely frozen. The ice was then removed by lyophilization to prepare CNF/Ti_3_CNT_x_ aerogels consisting of vertical pore morphology. Using the same procedure, vertically porous cellulose nanofibers/graphene oxide were prepared and then annealed under argon atmosphere to prepare CNF/rGO hybrid aerogels. During the annealing process, the oxygen-containing functional groups bound to the GO nanosheets inside the hybrid aerogel were removed, and then rGO nanosheets were obtained. A series of CNF/Ti_3_CNT_x_ aerogels with different contents were prepared and labeled as 2% CNF/T11, 5% CNF/T11, 8% CNF/T11% and 10% CNF/T11 according to the weight content of CNF, a macroscopic photograph of aerogel electrode are shown in Supplementary Figure S1, respectively.

**FIGURE 2:**
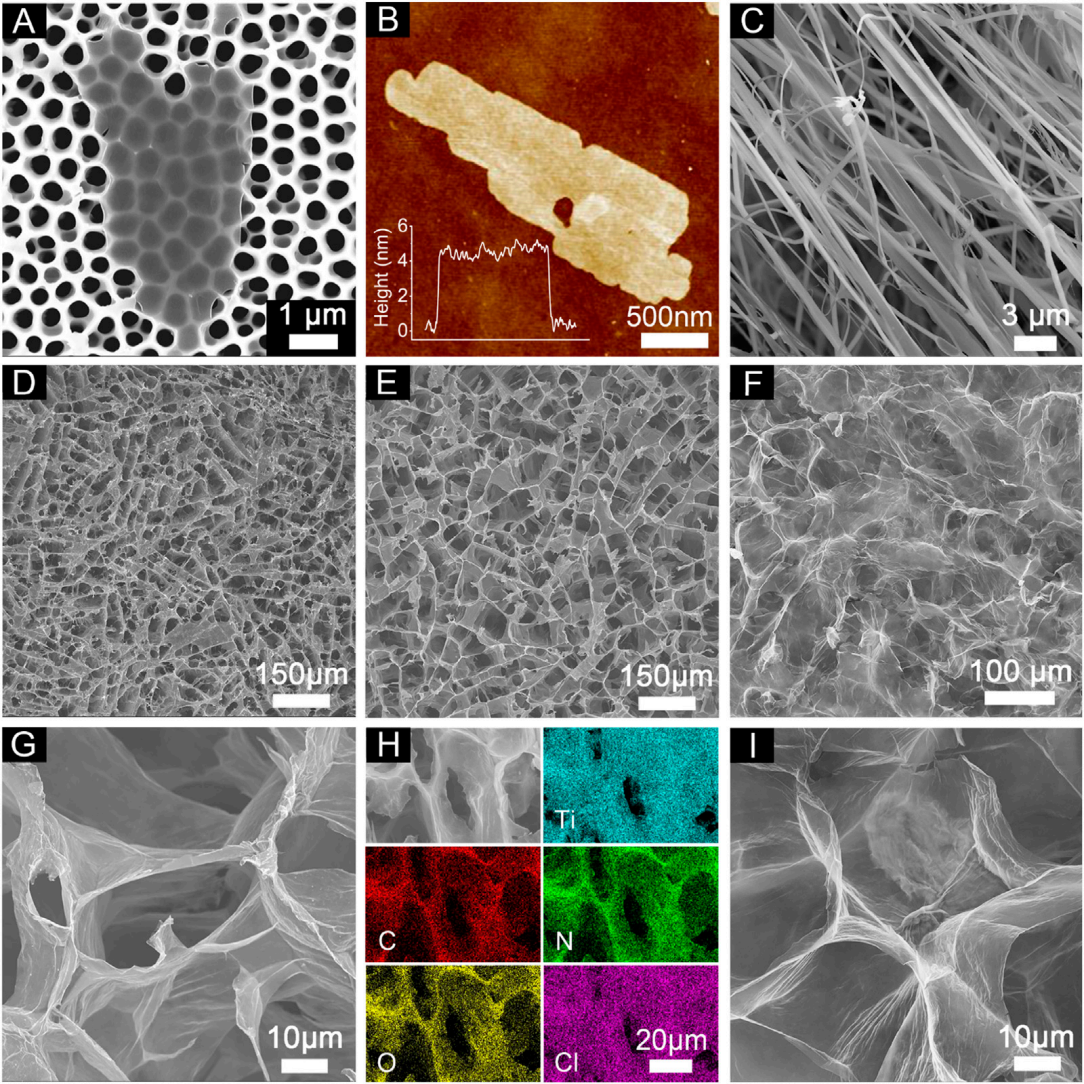
**(A)** SEM and **(B)** AFM images of the few-layer Ti_3_CNT_x_ nanosheets. **(C)** SEM image of the CNF. Top-view SEM images of the **(D)** 2% and **(E)** 5% CNF/Ti_3_CNT_x_ hybrid aerogels. **(F)** Top-view SEM image of the CNF/rGO hybrid aerogels. **(G, I)** The high-magnification SEM images of **(E, F)**, respectively. **(H)** Top-view EDS mapping images of the 5% CNF/Ti_3_CNT_x_ hybrid aerogels.

The morphology and microstructure of the prepared nanosheets, films and aerogels were investigated by scanning electron microscopy (SEM) and atomic force microscopy (AFM). As shown in Figure 2A, the size of the prepared few-layer Ti_3_CNT_x_ nanosheets is a few micrometers, which is favorable for the next step of aerogel assembly. As shown in Figure 2B, the AFM results further verified that the thickness of the few-layer Ti_3_CNT_x_ nanosheets was about 4.1 nm, and the prepared CNFs, as shown in Figure 2C, were distributed in linear spatial parallelism or intersections, which assisted in the construction of the aerogel mesh structure. The top-view SEM images of 2% CNF/T11, 5% CNF/T11, respectively, in Figures 2D,E, both clearly show that abundant vertical channels were formed in the hybrid aerogel, which would shorten the ion transport path and promote the ion diffusion rate. Comparing the top-view SEM images of aerogels with different contents (Figure 2D, E; Supplementary Figure S2), the vertical channel structure depends on the ratio of CNF and Ti_3_CNT_x_. As the mass ratio of CNF increases, more Ti_3_CNT_x_ nanosheets are conditioned by CNF, and the CNF/Ti_3_CNT_x_ hybrid aerogel has an increasingly open and ordered porous structure. Figures 2G,H show the high magnification images and EDS elemental maps of 5% CNF/T11, which clearly show the vertically aligned nanosheets and the corresponding vertical channels. The top-view SEM images and high-magnification images of 5% CNF/rGO, shown in Figures 2F,I, reveal that the rGO aerogel exhibits a more open and porous structure. The reason for the batter ordering may be that the CNF has a better binding force with the functional groups on the surface of Ti_3_CNT_x_, such as −OH, and then the auxiliary regulation of CNF on the Ti_3_CNT_x_ aerogel is more obvious. The surface of cellulose nanofibers prepared by TEMPO oxidation is rich in hydroxyl functional groups (Liu et al., 2023). And according to the FT-IR results, Ti_3_CNT_x_ also generated a large number of hydroxyl functional groups on the surface during the preparation process (Supplementary Figure S5). This suggests that CNF can bind to the Ti_3_CNT_x_ surface through a large number of hydrogen bonds, making the modulation of the Ti_3_CNT_x_ aerogel by CNF more pronounced. We visualize the construction of open and ordered vertical channels in the Ti_3_CNT_x_, rGO aerogels, which provide micrometer-level pathways for ion transport, facilitating faster diffusion of the electrolyte into the material and fully engaging in efficient charge transfer with the surface active sites to enhance the electrochemical energy storage kinetics.

**FIGURE 3:**
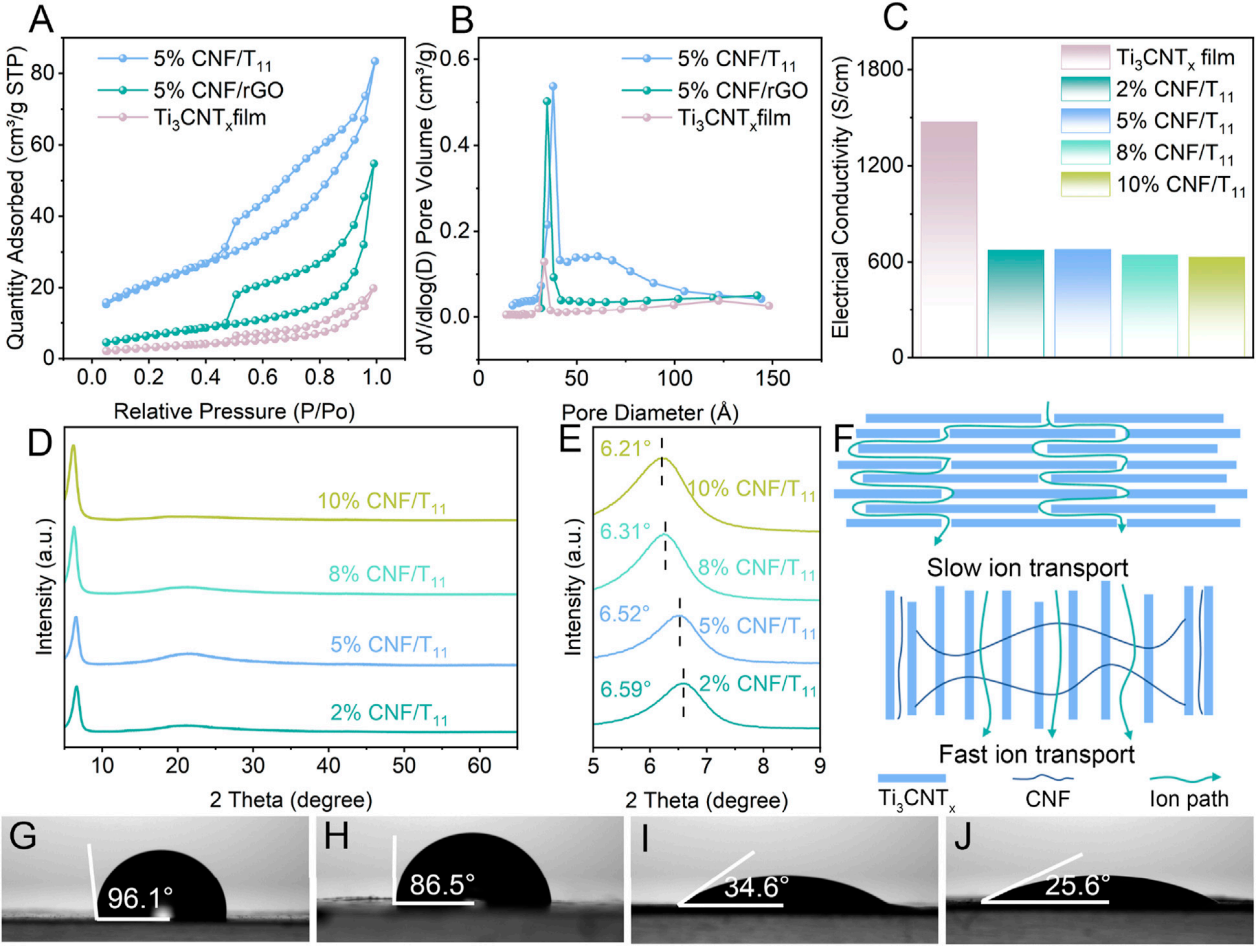
**(A)** N_2_ adsorption–desorption isotherms and **(B)** Pore size distribution of Ti_3_CNT_x_, 5% CNF/rGO and 5% CNF/Ti_3_CNT_x_ hybrid aerogels. **(C)** Electrical conductivity of Ti_3_CNT_x_ and CNF/Ti_3_CNT_x_ hybrid aerogels. **(D, E)** XRD patterns of Ti_3_CNT_x_ and CNF/Ti_3_CNT_x_ hybrid aerogels. **(F)** The schematic illustration of the ion pathway optimization in stacked Ti_3_CNT_x_ film comparing with the hybrid aerogel. **(G–J)** The electrolyte contact angle of the stacked rGO, 3D Ti_3_CNT_x_ film, CNF/rGO and CNF/Ti_3_CNT_x_ hybrid aerogels.

Adjacent 2D nanosheets are usually prone to restacking under van der Waals interactions, which hinders ion diffusion and reduces the available active surface. Therefore, we further investigated the formation of nano-channels by CNF insertion between nanosheet layers. Compared with the pristine Ti_3_CNT_x_ films, the 5% CNF/T11% and 5% CNF/rGO aerogels, have a porous structure and correspondingly higher specific surface area (Figure 3A). Due to the agglomeration of the nanosheets, the specific surface area decreases significantly. Therefore, the pore structure and interlayer spacing are the main factors affecting the specific surface area of porous hybridized aerogels. The intercalated CNF can effectively inhibit the agglomeration of nanosheets and expand the interlayer spacing, thus increasing the specific surface area of the hybrid aerogel, which is conducive to the realization of its excellent capacitive performance. And according to the main distribution of pore sizes of nanopores (Figure 3B) showed that the pore sizes of Ti_3_CNT_x_ films, 5% CNF/T11% and 5% CNF/rGO aerogels were increasing sequentially, which further confirmed that CNF intercalation enlarged the interlayer nanochannels of the 2D nanosheets and the better modulation effect of CNF on the Ti_3_CNT_x_ nanosheets in line with the results of SEM. We employed TGA analysis in the range of 30°C–600°C to elucidate the compositions of CNF/Ti_3_CNT_x_ and CNF/rGO hybrid aerogels, as shown in Supplementary Figure S6 rGO results showed only about 0.3% mass change throughout the temperature range which indicates that our reduction process has been very thorough, while the curve from 5% CNF/rGO shows that the depolymerization, dehydration, and decomposition of the glycosidic units (Rodríguez-Ramírez et al., 2022) in CNF from about 260°C onwards account for 4.5% of the mass fraction, which is comparable to our experimental setup of adding 5%, and the remaining 0.5% should be the decomposition product. The results for Ti_3_CNT_x_ show that there is about 1.2% mass change throughout the temperature range, which should partly come from the loss of H_2_O and surface hydroxyl groups inside Ti_3_CNT_x_, and also about 4.6% CNF is decomposed with some decomposition products retained, which is comparable to our experimental setting of 5%. In addition, consistent with the results of most studies, the construction of porous structure will inevitably lead to a decrease in conductivity due to the presence of spatial impedance, as shown in Figure 3C, the conductivity of the aerogel is generally decreasing with the increase in the proportion of added CNF due to the inability of electron transfer between the nanosheets and the non-conductivity of the CNF itself. However, the excellent conductivity of Ti_3_CNT_x_ with metal grade allows the designed aerogel to maintain a high conductivity, which ensures the fast charge transfer condition of the electrode (Zhao et al., 2017). XRD spectra were recorded to study the exact layer spacing and phase transitions as shown in Supplementary Figure S3; Figure 3D, E. The XRD spectra of Ti_3_CNT_x_ are shown in Supplementary Figure S3 and Figure 3D, E. The characteristic (002) peak of Ti_3_AlCN at about 10.0° shifted to a lower angle at 6.66°, verifying the successful etching of the aluminum layer from Ti_3_AlCN. With the increase of CNF mass ratio in the hybrid aerogel, the position of the (002) peak shifts from 6.66° for Ti_3_CNT_x_ film to 6.21°for 10% CNF/T11 with a significant increase in intensity, which demonstrates that the cellulose as a nanospace medium efficiently prevents the stacking of the nanosheets and enhances the ordering of the Ti_3_CNT_x_ arrangement. In addition, Supplementary Figure S3 shows the XRD patterns of CNF and rGO, which are consistent with previous reports. We performed Raman tests on 5% CNF/GO and 5% CNF/rGO, the results of which are shown in Supplementary Figure S7. The Raman survey spectra show a significant increase in the intensity ratio of the D-band (defective vibrations) to the G-band (graphitic carbon vibrations) from graphene oxide (0.89) to reduced graphene oxide (1.04), suggesting that defect-rich rGO is formed. In addition, the reduced graphene oxide showed a distinct 2D peak with a half-height width of 1.01 cm^−1^ and an I_G_/I_2D_ value of 0.24, which further indicates that the graphene oxide was successfully reduced and is a monolayer of reduced graphene oxide. In summary, nanochannel-coupled vertical porous structures have been successfully constructed for optimizing ion transport paths, enhancing charge/ion transfer/diffusion kinetics, and forming robust and efficient ASC electrodes. As shown in Figure 3F which displays the structural and electrolyte-ion path differences between the pristine film and the optimized electrodes. Furthermore, the electrolyte contact angles of rGO and Ti_3_CNT_x_ dense films were 96.1° and 86.5°, respectively (Figures 3G,H). Nanochannel-coupled vertically porous CNF/T11 and CNF/rGO aerogels improve the surface porosity and hygroscopicity. Due to capillarity and hygroscopicity, the contact angles of the electrolytes of CNF/rGO and CNF/T11 were as low as 34.6° and 25.6°, respectively (Figures 3I,J), which resulted in good electrolyte storage in the electrodes. In order to confirm the optimization of the mechanical properties of aerogels by CNF, we tested the stress-strain curves of aerogels and the electrochemical properties under different strains (Supplementary Figure S8). The results show that the addition of cellulose nanofibers enables the aerogel to withstand greater stress and strain, which confirms the significant enhancement of the mechanical properties of the aerogel by CNF. In addition 5% CNF/T11 aerogel was able to produce smaller strains than 5% CNF/rGO aerogel under great stresses, which further this suggests that CNF has a better modulation of the structure of Ti_3_CNT_x_. Finally, we evaluated the electrochemical performance of the electrodes after generating the maximum strain, and the CV curves of the electrodes under the maximum strain remained basically unchanged when the scan rate was 20 mV s^−1^, and the CV curves obtained also overlapped with those of the unstrained electrodes in terms of the peak values. This indicates that the CNF-optimized aerogel electrode has excellent mechanical stability and electrochemical performance.

### 3.2 The electrochemical performance of CNF/Ti_3_CNT_x_ anode

**FIGURE 4:**
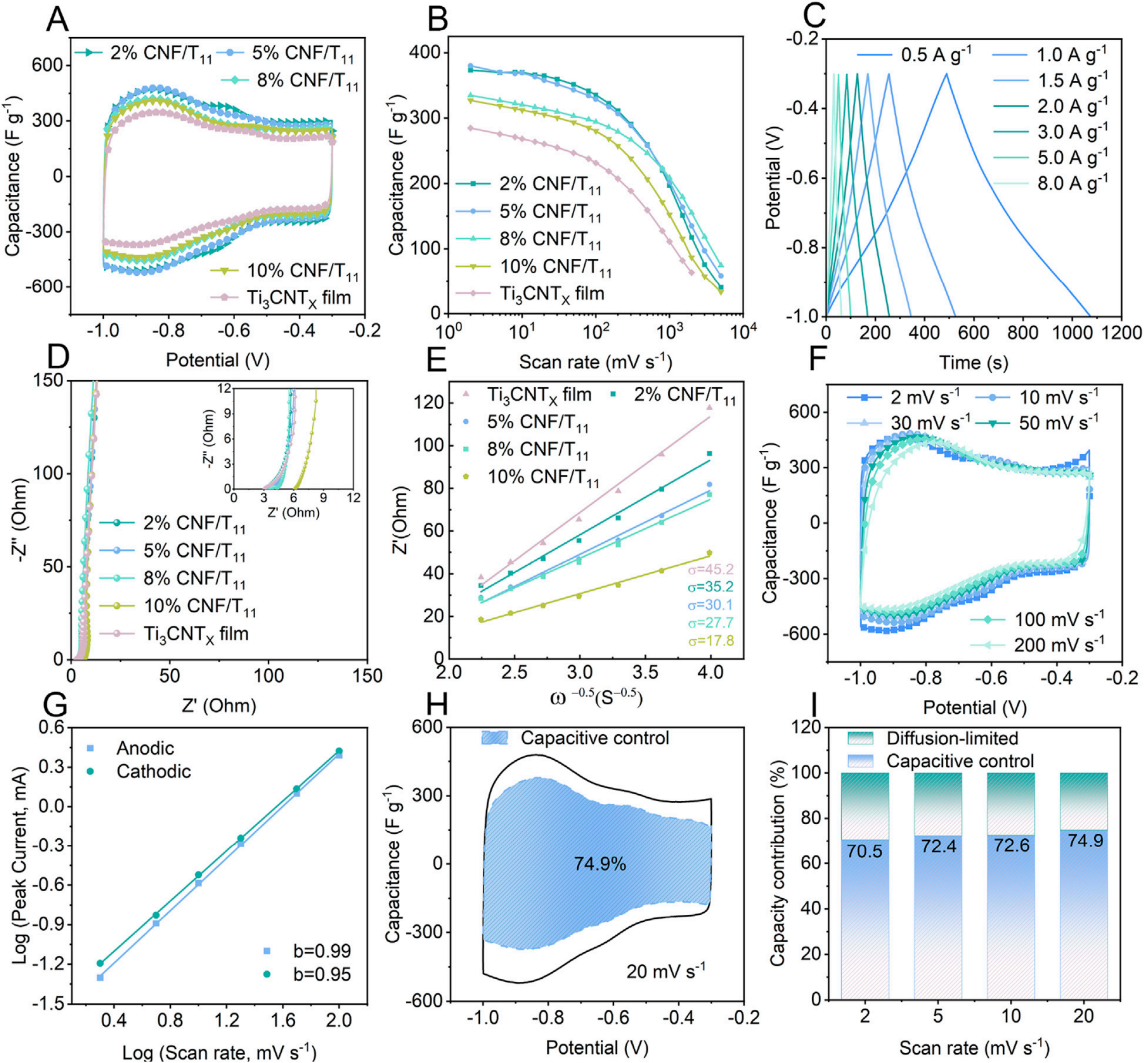
Electrochemical performances of the fabricated hybrid aerogels. **(A)** CV curves of Ti_3_CNT_x_ film and CNF/Ti_3_CNT_x_ composite aerogels with different CNF contents at 20 mV s^−1^. **(B)** Dependence of the gravimetric capacitance of Ti_3_CNT_x_ film and CNF/Ti_3_CNT_x_ composite aerogels on different scan rates. **(C)** GCD curves of 5% CNF/Ti_3_CNT_x_ composite aerogel at different current densities. **(D)** Nyquist plots of the Ti_3_CNT_x_ film and CNF/Ti_3_CNT_x_ composite aerogels **(E)** and the liner relation of ω^−0.5^ vs Z’. **(F)** CV curves of 5% CNF/Ti_3_CNT_x_ at different scan rates. **(G)** The power-law dependence of the response current on scan rates. **(H)** Capacitance contribution of 5% CNF/Ti_3_CNT_x_ at 20 mV s^−1^. **(I)** Capacitive and diffusion-controlled contributions to the total capacitance of 5% CNF/Ti_3_CNT_x_ at different scan rates.

To explore the energy storage advantages of CNF/Ti_3_CNT_x_ hybrid aerogels with vertical porous structure, we performed electrochemical tests using a three-electrode cell in a 2 M sulfuric acid electrolyte. Figure 4A shows the cyclic voltammetry (CV) curves of the hybrid aerogels with different CNF contents. Two pairs of obvious redox peaks can be observed around −0.62 V and −0.85 V, demonstrating the existence of Faraday pseudocapacitance on the surface of Ti_3_CNT_x_ nanosheets involved in reversible redox reactions. When the scanning potential polarity is changed, the current polarity is immediately reversed, which indicates that the vertically porous CNF/Ti_3_CNT_x_ hybrid aerogel has a high electron transfer rate and ion diffusion rate. Among the CNF/Ti_3_CNT_x_ hybrid aerogels and Ti_3_CNT_x_ films, 2% CNF/T11 has the largest integrated CV area, thereby indicating that the 2% CNF/T11 sample also exhibits the highest capacitive performance. 5% CNF/T11 capacity slightly decreased, while the other electrodes show a significant decrease in capacity. This is due to the fact that the CNF intercalation and vertical porous structure inhibit the nanosheet stacking and provide more redox active sites, whereas the CNF itself does not have energy storage activity, and an excess of it rather reduces the overall electrode mass specific capacitance. Compared with the Ti_3_CNT_x_ film, the rate capability of the hybrid aerogel is also significantly improved due to the unique nano-channel-coupled vertical porous structure, which improves the ion diffusion kinetics, and the mass specific capacitance of the 5% CNF/T11 electrode is as high as 380 F g^−1^ at 2 mV s^−1^. A high capacitance of 203 F g^−1^ is maintained even at 1,000 mV s^−1^ (Figure 4B). The GCD curve of 5% CNF/T11 (Figure 4C) shows an approximate triangle, indicating the capacitive nature of electrochemical energy storage, which is consistent with the CV results in Figure 4A.

To further elucidate the energy storage kinetics of the hybrid aerogel, electrochemical impedance spectroscopy (EIS) was performed to analyze the ion diffusion kinetics in the frequency range of 100 kHz to 0.01 Hz. The equivalent circuit shown in circuit diagram (Supplementary Figure S9) was used to fit the Nyquist diagram of the CNF/Ti_3_CNT_x_ hybrid aerogel in Figure 4D. The equivalent series resistance gradually increases with increasing mass ratio of cellulose nanofibers due to the fact that CNFs are inherently non-conductive, which is consistent with the conductivity test results. It is worth noting that, according to the ion diffusion coefficient equation, the Warburg coefficient σ indicates the rapid diffusion process of electrolyte ions, which is inversely related to the diffusion coefficient D (Ruan et al., 2023). The calculated σ values of the CNF/Ti_3_CNT_x_ hybrid aerogels are significantly smaller than those of the Ti_3_CNT_x_ films and decrease with the increase of the specific gravity of CNF, which means that the diffusion coefficient gradually increases and the diffusion process is accelerated, which further proves that nano-channel-coupled aerogels with vertical porous structure can improve the ion diffusion kinetics. To investigate the ionic reaction kinetics of 5% CNF/Ti_3_CNT_x_ aerogel, we analyzed the results of CVs at different scan rates (Figure 4F). The charge storage kinetics were elucidated by calculating the slope ( b ) of log ( i ) versus log ( v ), where i corresponds to the peak current and v corresponds to the scan rate. b values distinguish diffusion-controlled processes ( b = 0.5) from surface-controlled processes ( b = 1). The b value for the 5% CNF/Ti_3_CNT_x_ anodic current is 0.99 (Figure 4G), demonstrating that the process is mainly controlled by the surface controlled kinetics. In addition, the capacitance-controlled and diffusion-controlled ratios at different scan rates can be further quantified by calculating $k_{1}$ and $k_{2}$ according to the following Equations 8, 9 (Wang et al., 2007):

$$I=av^{b}$$ (8)

$$I\left(v\right)=k_{1}v+k_{2}v^{0.5}$$ (9)

Therefore, according to the calculation, the capacitive contribution will increase from 70.5% at 2 mV to 74.9% at 20 mV s^−1^ (Figures 4H,I). This indicates that the charge storage process in 5% CNF/Ti_3_CNT_x_ is essentially dominated by the surface Faraday pseudocapacitance behavior rather than by diffusion-controlled cell-type processes. This facilitates the realization of excellent cycling stability and ionic reaction kinetics.

### 3.3 The electrochemical performance of CNF/rGO cathode

To assemble ASCs with CNF/Ti_3_CNT_x_ aerogel anode pairing and to demonstrate the feasibility of CNF/rGO aerogels. We used the same method to synthesize nanochannel-coupled vertically porous redox graphene aerogel (5% CNF/rGO). 5% CNF/rGO possesses a high positive potential window (−0.4–0.5 V) (Supplementary Figure S10A), which is capable of broadening the voltage window of the device and realizing the increase of the energy density in assembling ASCs. A pair of redox peaks appeared near −0.05 V in the variable-rate CV (Supplementary Figure S10A), which was attributed to the increase of redox active sites after redox, thus introducing pseudo-capacitive energy storage in graphene aerogel. The approximate linear GCD curve (Supplementary Figure S10B) is consistent with the CV results. The CNF/rGO aerogel with smaller equivalent internal resistance and Warburg factor σ (Supplementary Figure S10C, D) was analyzed based on the EIS data, indicating that the CNF/rGO aerogel also possesses a better electrical conductivity and a fast ion diffusion rate, which can match with the CNF/rGO aerogel in terms of energy storage kinetics. In addition, the higher mass specific capacitance and excellent multiplicity performance of CNF/rGO aerogel also determine that it can be well matched with CNF/Ti_3_CNT_x_ (Supplementary Figure S10E).

### 3.4 The electrochemical performance of CNF/rGO//CNF/Ti_3_CNT_x_ ASC

**FIGURE 5:**
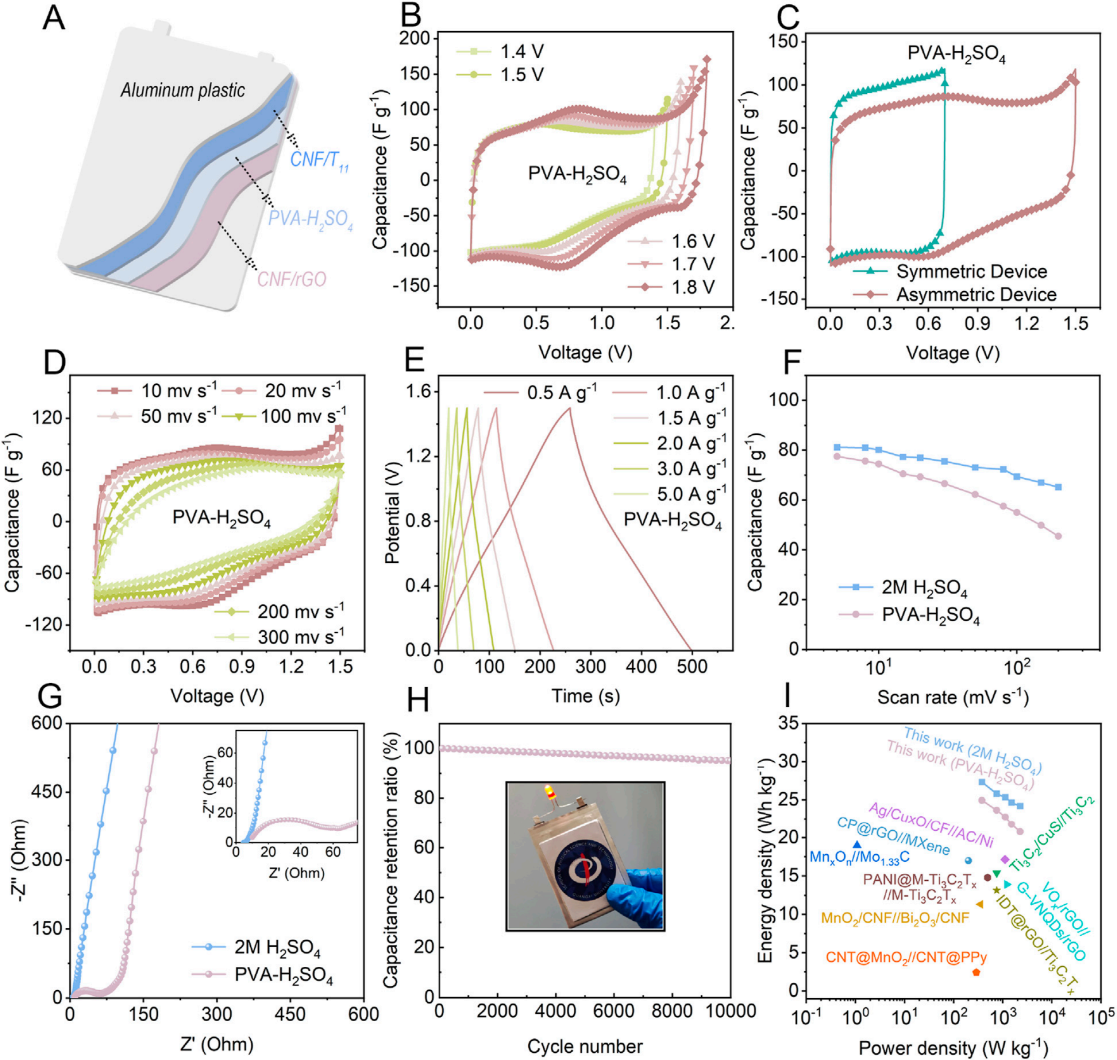
Electrochemical performance of the 5% CNF/Ti_3_CNT_x_//5% CNF/rGO ASC devices. **(A)** Schematic diagram of the all-solid-state ASC. **(B)** CV curves of the ASCs within different voltage ranges at 20 mV s^−1^. **(C)** CV curves of the ASCs and SSCs with PVA-H_2_SO_4_ gel. **(D)** CV curves and **(E)** GCD curves of the ASCs with PVA-H_2_SO_4_ gel. **(F)** Rate performance of the ASCs at different scan rates. **(G)** Nyquist plots of the ASCs. **(H)** The capacitance retention of the all-solid-state supercapacitors after 10,000 cycles at 5 A g^−1^, the inset in **(H)** is the photograph of the all-solid-state ASC with a single LED lit. **(I)** Ragone plots of the fabricated ASCs and previously reported ASC.s.

To demonstrate the actual charge storage performance of CNF/Ti_3_CNT_x_ and CNF/rGO hybrid aerogels prepared using the proposed method, we assembled asymmetric supercapacitors (ASCs) with 5% CNF/Ti_3_CNT_x_ as anode and 5% CNF/rGO as cathode in either 2 M H_2_SO_4_ electrolyte or PVA-H_2_SO_4_ gels (Figure 5A). To better highlight the advantages of asymmetric supercapacitors, we also assembled and tested symmetric supercapacitors (SSCs) for comparison (Supplementary Figure S11). Compared with the lower stable operating voltage of only 0.7 V at different scan rates in SSCs (Supplementary Figure S11), the CV curves of the ASCs in both electrolytes with different voltage windows verified that the stable operating voltage window can be extended up to 1.5 V and even as high as 1.8 V before polarization occurs (Figures 5B,C; Supplementary Figure S11A). The higher operating voltage window range is favorable for improving the energy density of asymmetric supercapacitors. In addition, in the CV curves at different rates of SSCs and ASCs (Figure 5D), SSCs show quasi-rectangular shape in all CV curves due to the adoption of the same energy storage characteristics of the symmetric electrodes, especially in the PVA-H_2_SO_4_ gels (Supplementary Figure S11C). And a pair of obvious redox peaks can be observed for ASCs under both electrolytes around 0.6 V, 0.7 V (Figure5D; Supplementary Figure S12B), respectively, which indicates that ASCs have more obvious pseudocapacitive energy storage characteristics, and are more capable of combining the energy storage characteristics of different electrodes under the effect of mutual compensation during the asymmetric electrode matching, confirming that the excellent gravimetric capacitance of ASCs partly originates from the more prominent pseudocapacitive characteristics. The corresponding GCDs (Figure 5E) show excellent reversibility and high Coulombic efficiency. Based on the CV calculated from the total weight of the two electrodes, the gravitational capacitance of ASCs in 2 M H_2_SO_4_ or PVA-H_2_SO_4_ gels reaches 81 and 77 F g^−1^, respectively. The calculated gravitational capacitance at different scanning rates is shown in Figure 5F, where the gravitational capacitance of the all-solid-state ASCs stays at about 72% of the initial value when the scanning rate is increased to 100 mV s^−1^. In addition, the charge transfer impedance in the PVA-H_2_SO_4_ gels increased due to the higher impedance of PVA, but the fast ion diffusion rate was maintained in the all-solid-state ASC (Figure 5G). In view of the importance of cycle life for ASC applications, the cycle life of ASC was tested accordingly. After 10,000 cycles at 5 A g^−1^, the all-solid-state ASC retained 95.1% of its initial capacitance (Figure 5H), showing remarkable reversibility and long-term stability. To investigate the reason for the electrode charge/discharge cycling stability, the SEM of the electrode after cycling was tested (Supplementary Figure S13), which shows that the electrode still maintains a stable vertical porous morphology, suggesting that the aerogel electrode can be well maintained mechanically stable during the cycling process with the assistance of CNF, and proving the positive effect of the structural design of nanochannel-coupled vertical porosity on the highly reversible cycling performance. The inset in Figure 5H shows the all-solid-state ASC with a single LED lit. The more active surfaces of the nanochannel-coupled vertically porous CNF/Ti_3_CNT_x_ and CNF/rGO hybrid aerogel ensured a high gravitational capacitance of the anode and the negative. Higher gravimetric capacitance of the assembled ASCs successfully broadens the device voltage, resulting in higher energy density of the ASCs, while the similar structure of the positive and negative electrodes endows them with fast charge storage kinetics, ensuring that the ASCs provide relatively high energy density even at high power density. The all-solid-state ASC device achieves a maximum energy density of 20.8 Wh kg^−1^ while maintaining a high power density of 2.3 kW kg^−1^, which is significantly better than the SSCs (Supplementary Figure S14). As shown in Figure 5I, the ASCs assembled in this work are competitive in terms of both high energy density and high power density, surpassing the recently reported ASCs (Zhao et al., 2015; Yan et al., 2017; Fan et al., 2018; Boota and Gogotsi, 2019; Guo et al., 2019; Kandambeth et al., 2020; Ghosh and Pumera, 2021; Wu et al., 2021; Wang et al., 2022). This further illustrates that designing nano-channel-coupled vertically porous CNF/Ti_3_CNT_x_ and CNF/rGO hybrid aerogels with matched structural and electrochemical properties can lead to the development of high-performance ASCs.

## 4 Conclusion

In summary, we have successfully constructed vertically porous CNF/Ti_3_CNT_x_ hybrid aerogels by a unidirectional bottom-up freezing method, in which the intercalated CNF cellulose nanofibers are shown to inhibit the re-stacking of Ti_3_CNT_x_ nanosheets, expand the interlayer nano-channels, improve the structural stability of the whole hybrid aerogel, and form a good electrolyte storage in the electrode. The formation of interlayer nano-channels increases the accessibility of electrochemically active sites on the Ti_3_CNT_x_ surface. In addition, the designed vertical porous structure effectively shortens the diffusion path of ions through the electrode. The structural design of nanochannel-coupled vertical porous enhances the charge/ion transfer/diffusion kinetics, enabling the electrode to perform excellent capacitive performance at high charge/discharge rates. The pairing of prepared graphene and MXene electrodes as cathode and anode of ASCs allows wider potential window, high rate charge storage kinetics, and high structural stability. The optimized electrochemical properties of the electrodes enabled the all-solid-state ASCs to achieve an excellent energy density of 20.8 Wh kg^−1^ at a high power density of 2.3 kW kg^−1^, high rate performance, and remarkable cycling stability (95.1% retention after 10,000 cycles). In this work, nanochannel-coupled vertical porous electrodes with matched structural and electrochemical properties were designed to provide an effective strategy for the development of ASCs and a means of advancing the rational design of optimized electrodes for advanced energy storage technologies.

## Data Availability

The original contributions presented in the study are included in the article/Supplementary Material, further inquiries can be directed to the corresponding author.
