# Supplementary material for: Vertical porous 1D/2D hybrid aerogels with highly matched charge storage performance for aqueous asymmetric supercapacitors
Source: Front Chem. 2025 Feb 28;13:1550285. doi: 10.3389/fchem.2025.1550285 (PMC11906718; doi:10.3389/fchem.2025.1550285)
Supplement: Supplementary file 1 [file DataSheet1.docx]

Supplementary Material

# Supplementary Figures

**
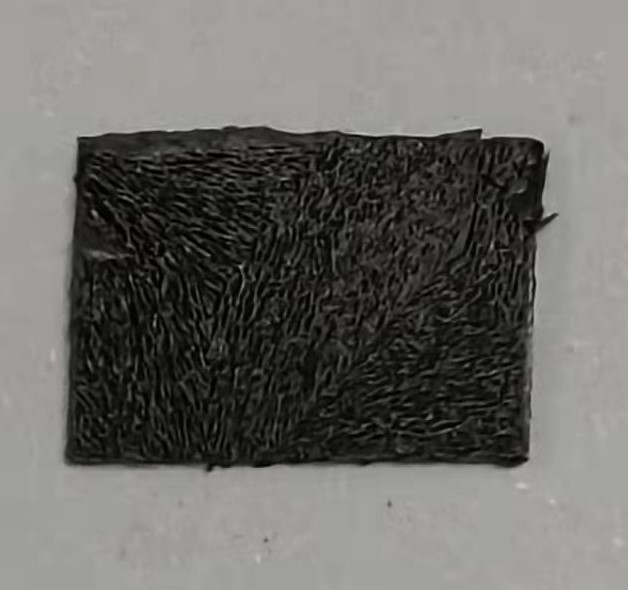
**

**Supplementary Figure 1.** Macroscopic photograph of the aerogel electrode.


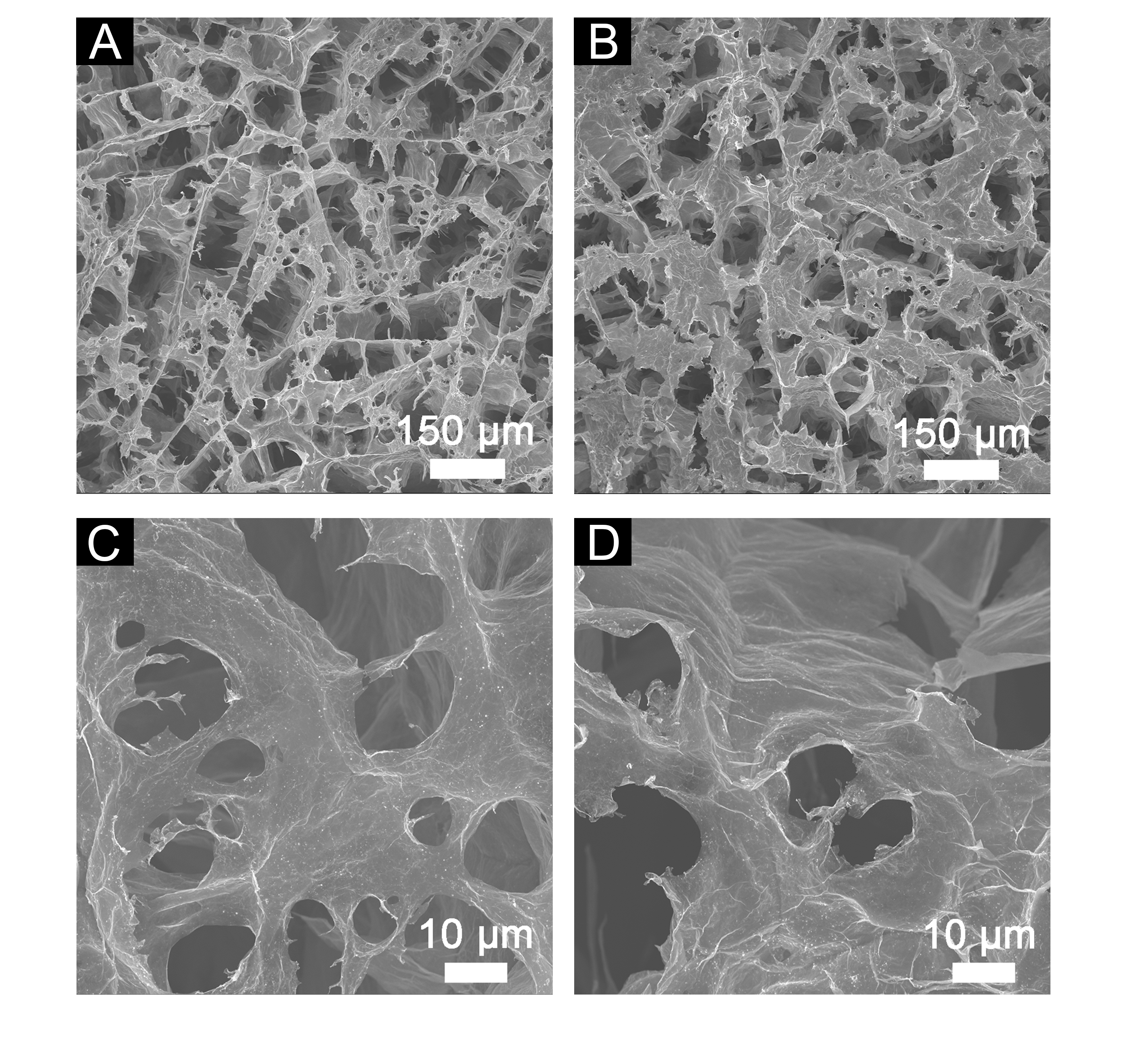


**Supplementary Figure 2.** Top-view SEM images of the (A) 8% and (B) 10% CNF/Ti_3_CNT_x_ hybrid aerogels. (C, D) the high-magnification SEM images of (A, B), respectively.


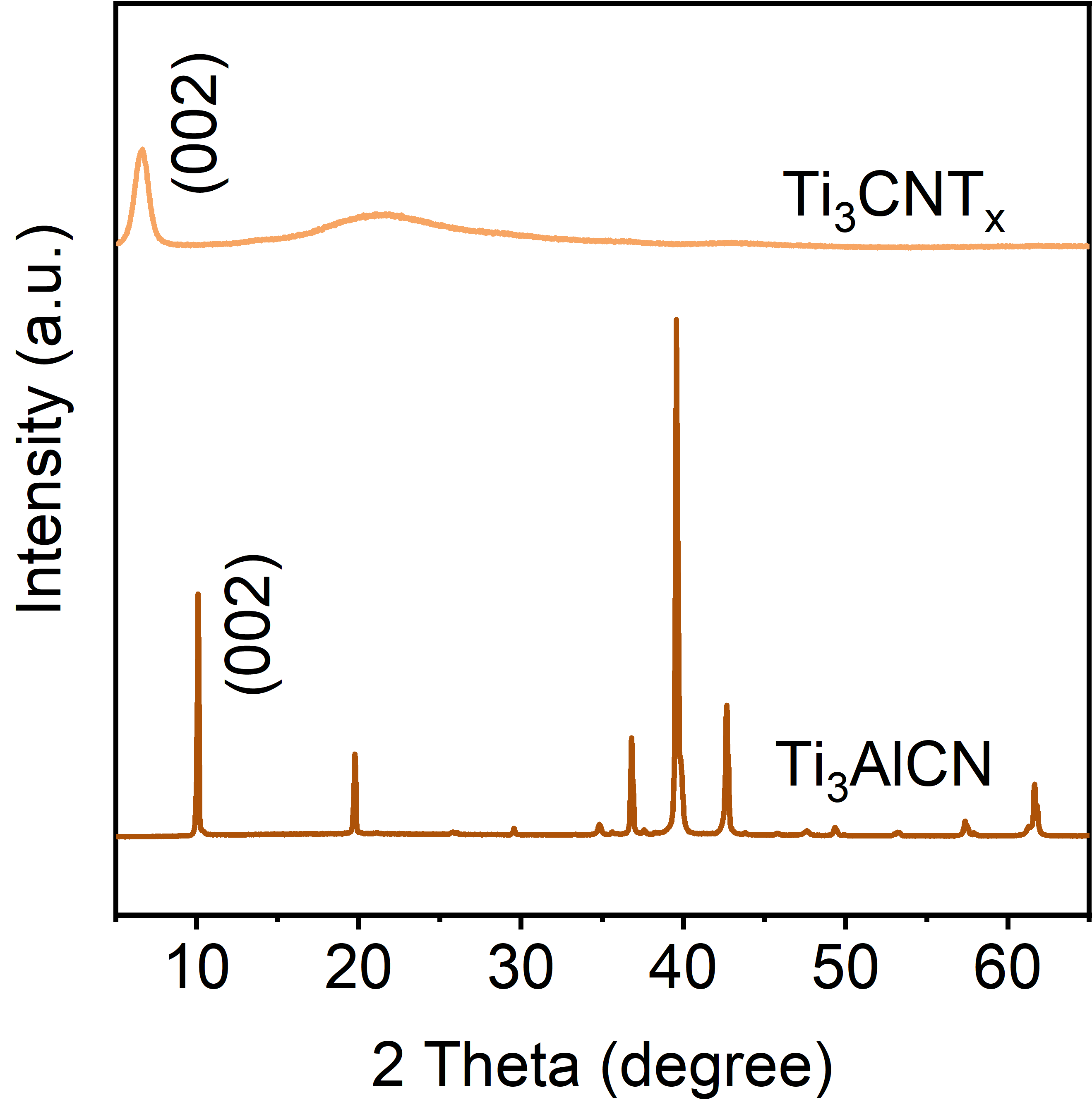


**Supplementary Figure 3.** XRD patterns of Ti_3_AlCN and the pure Ti_3_CNT_x_ film.


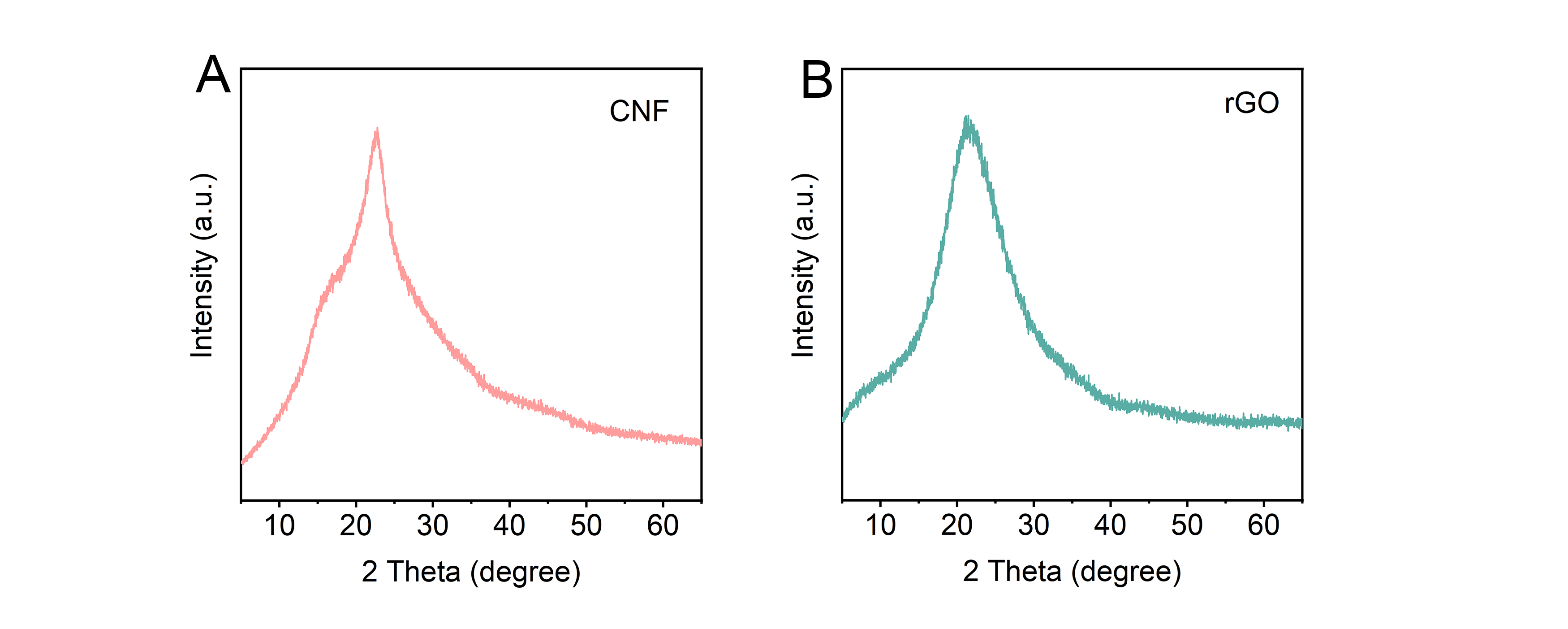


**Supplementary Figure 4.** (A) XRD patterns of CNF and (B) XRD patterns of rGO.


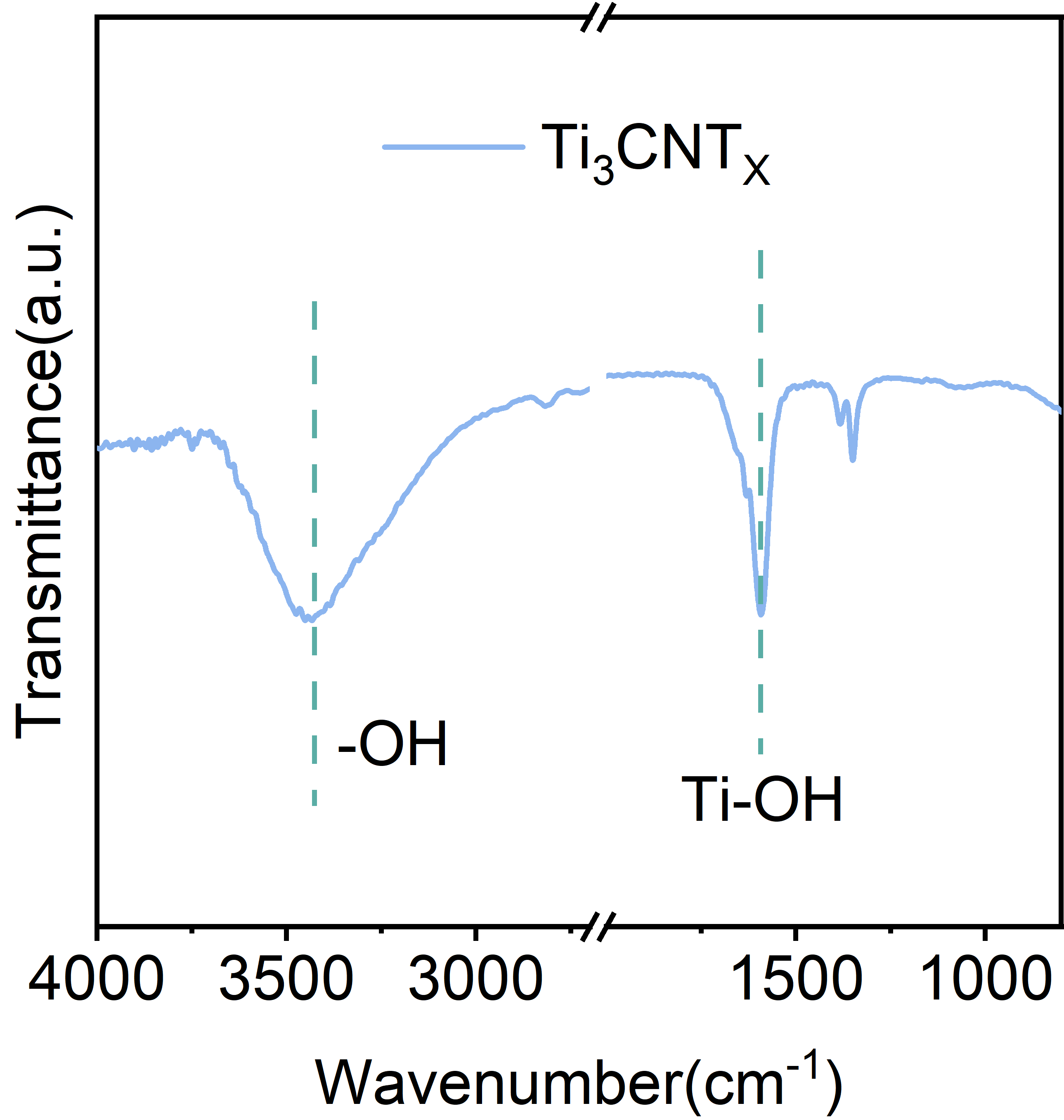


**Supplementary Figure 5.** FT-IR spectrum of Ti_3_CNT_x_.


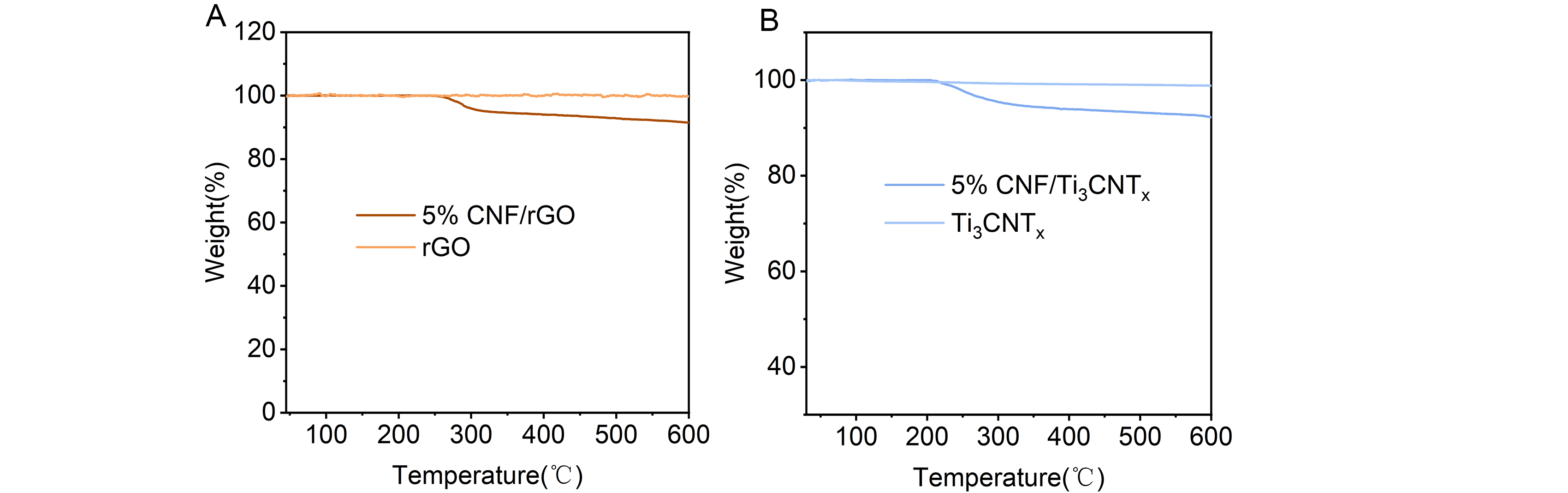


**Supplementary Figure 6.** Thermogravimetric analysis for 5% CNF/Ti_3_CNT_x_, 5% CNF/rGO, Ti_3_CNT_x_, and rGO.


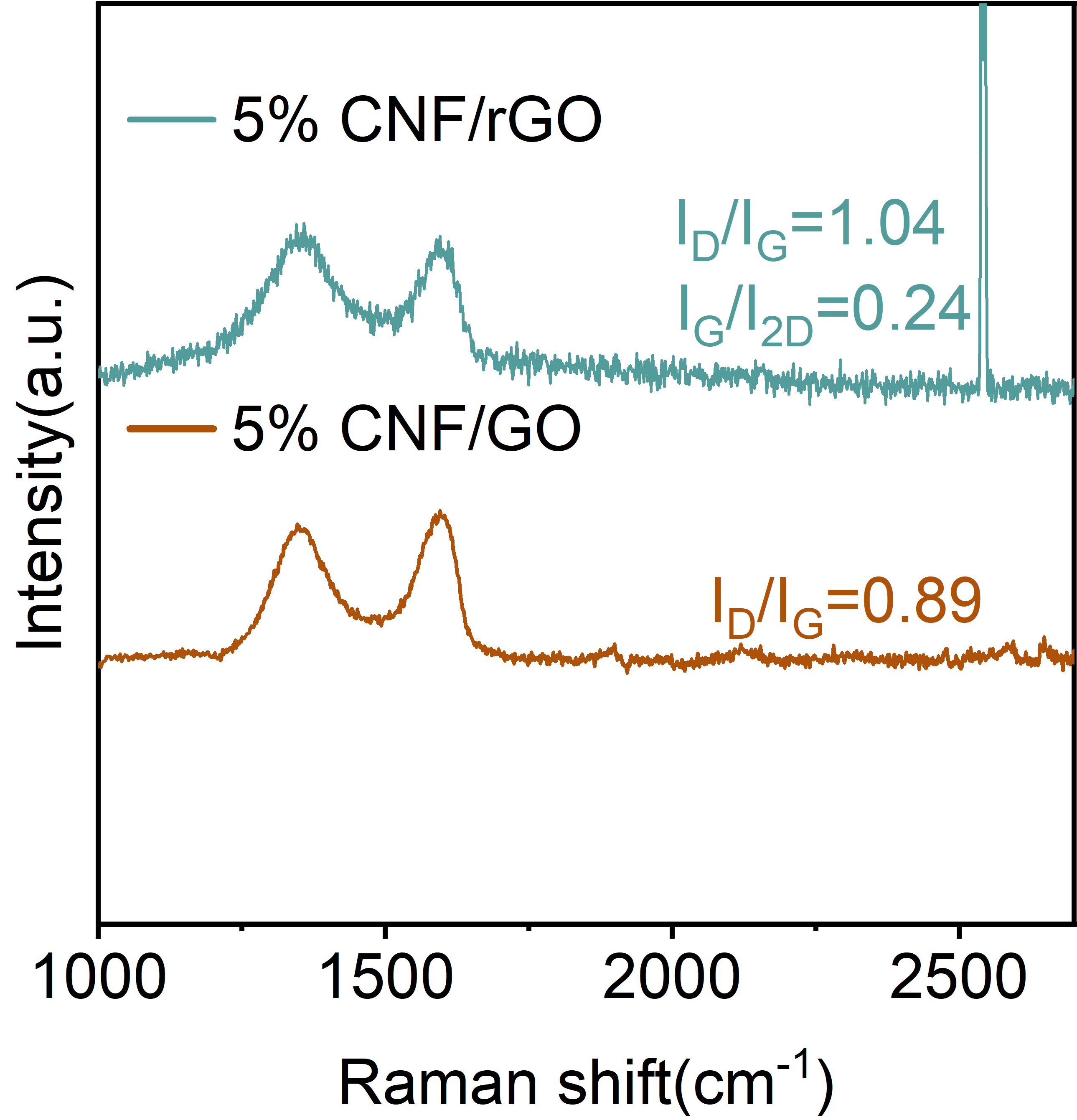


**Supplementary Figure 7.** Raman patterns of 5% CNF/GO and 5% CNF/rGO.


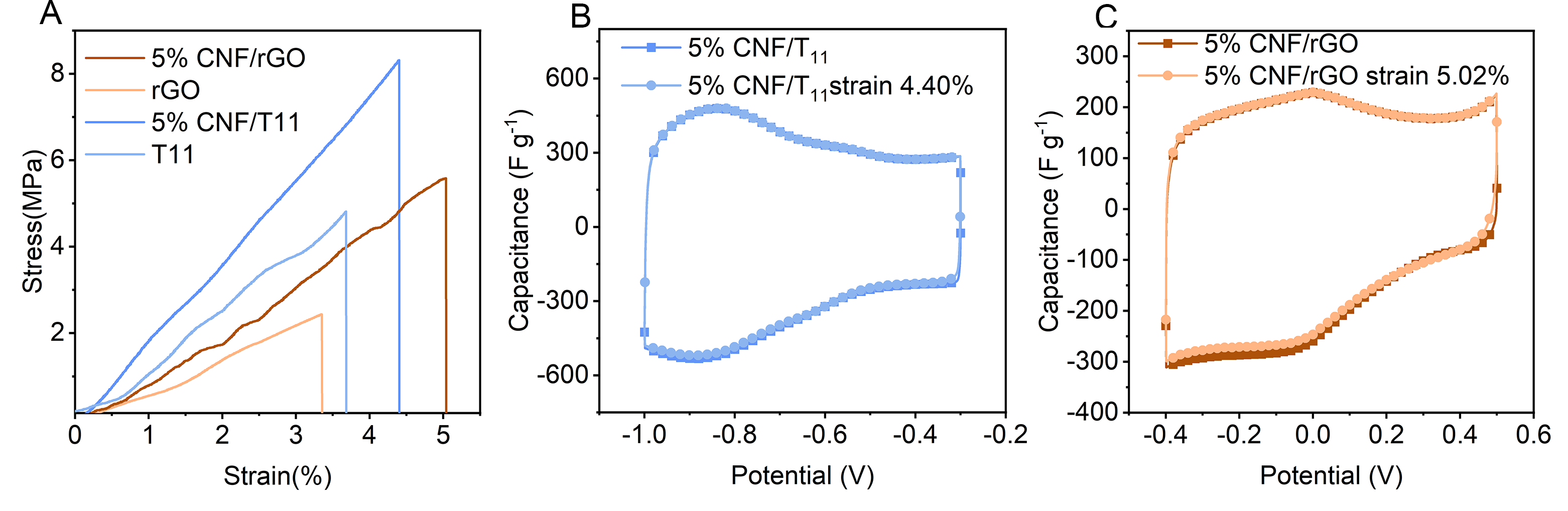


**Supplementary Figure 8.** (A)Stress-strain curves for the aerogels. (B,C) CV curves for different strains of aerogels tested at 20 mV s^-1^.


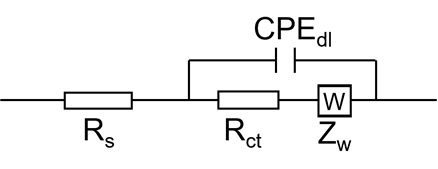


**Supplementary Figure 9.** The equivalent circuit used for EIS fitting.


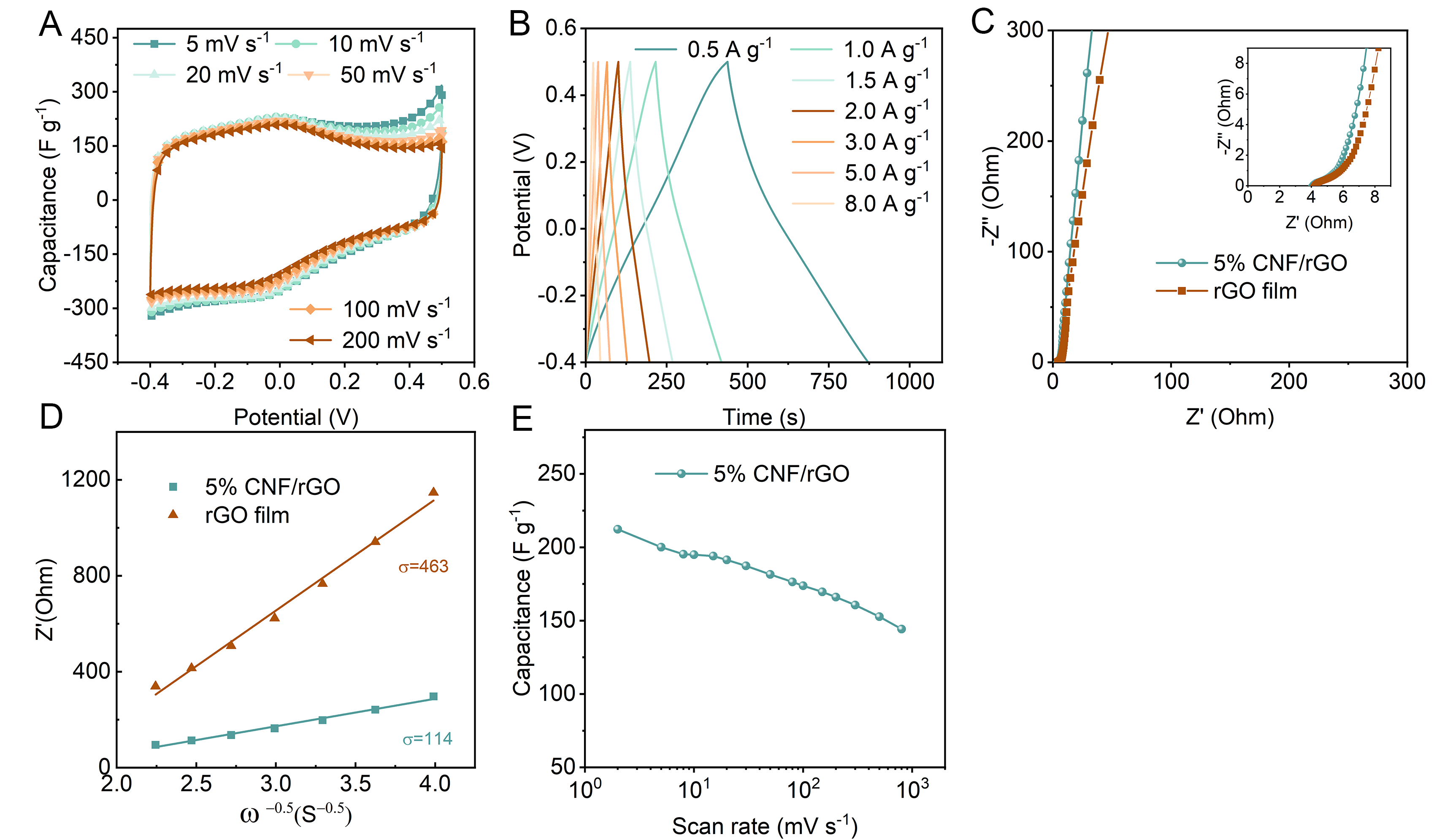


**Supplementary Figure 10.** Electrochemical performances of the fabricated hybrid aerogels. (A) CV curves of 5% CNF/rGO at different scan rates. (B) GCD curves of 5% CNF/rGO composite aerogel at different current densities. (C) Nyquist plots of the rGO film and CNF/rGO composite aerogel (D) and the liner relation of ω^−0.5^ vs. Z’. (E) Dependence of the gravimetric capacitance of 5% CNF/rGO composite aerogel on different scan rates.


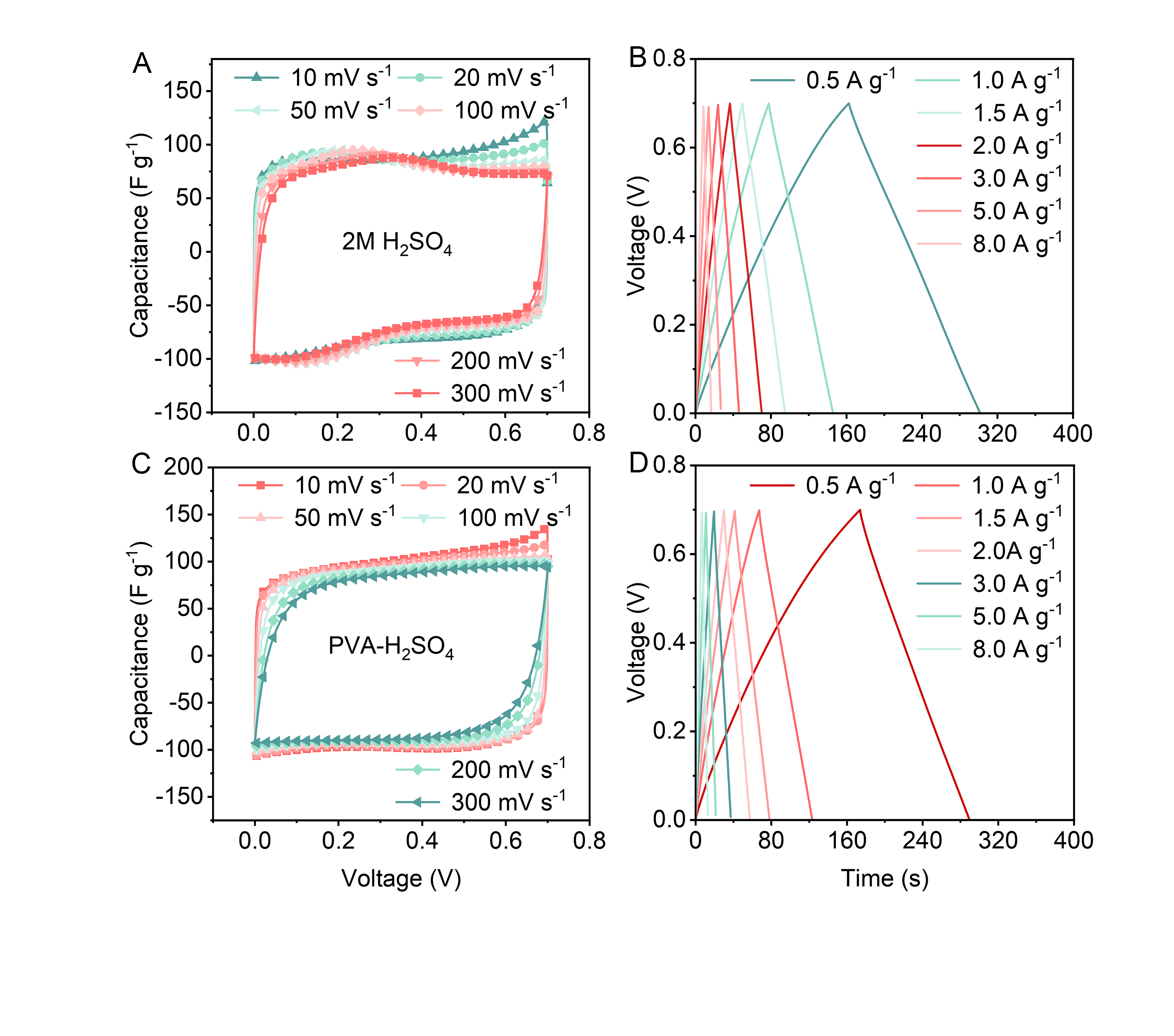


**Supplementary Figure 11.** (A) CV curves and (B) GCD curves of the 5% CNF/Ti_3_CNT_x_ SSCs in 2 M H_2_SO_4_ electrolyte. (C) CV curves and (D) GCD curves of the SSCs with PVA-H_2_SO_4_ gel.


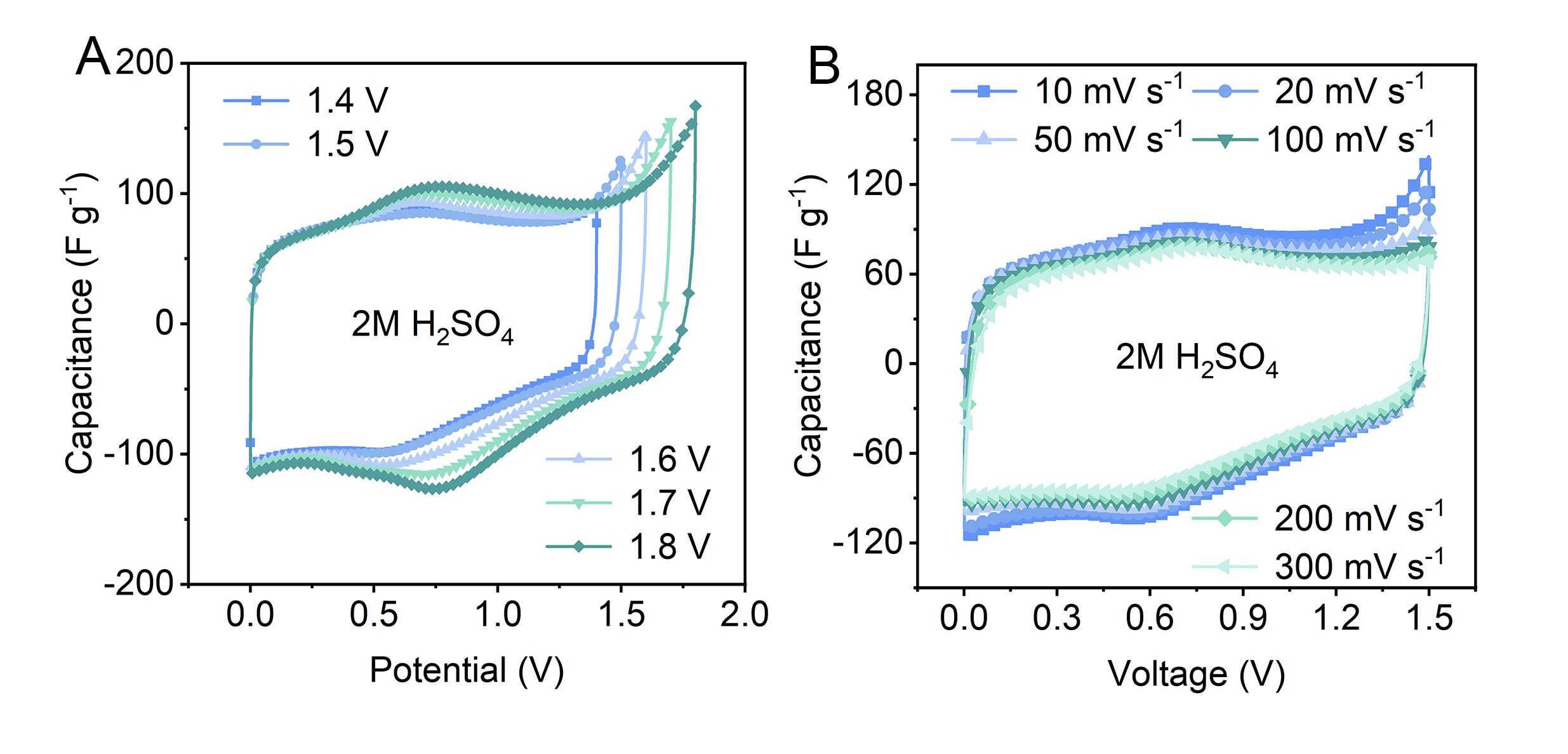


**Supplementary Figure 12.** (A) CV curves of the ASCs within different voltage ranges at 20 mV s^−1^ in 2 M H_2_SO_4_ electrolyte. (B) CV curves of the ASCs in 2 M H_2_SO_4_ electrolyte.


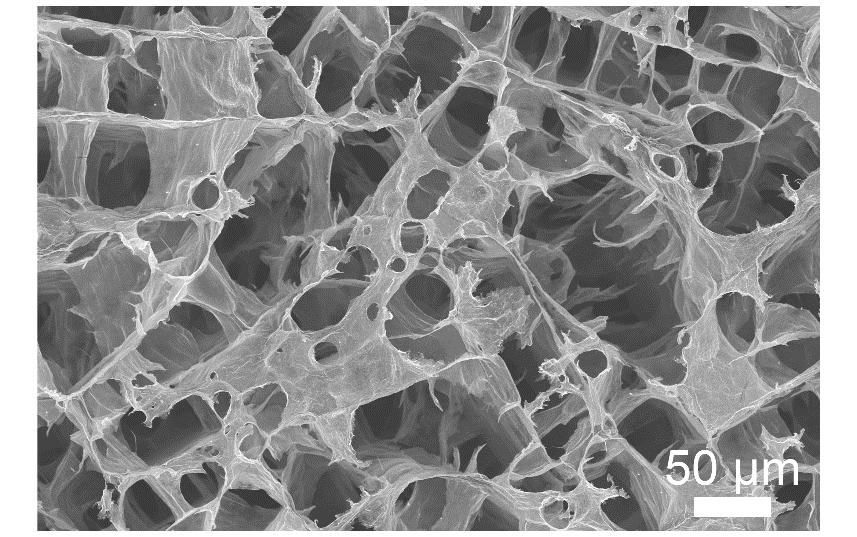


**Supplementary Figure 13.** Top-view SEM image of the 5% CNF/Ti_3_CNT_x_ hybrid aerogels after charge/discharge cycles.

**
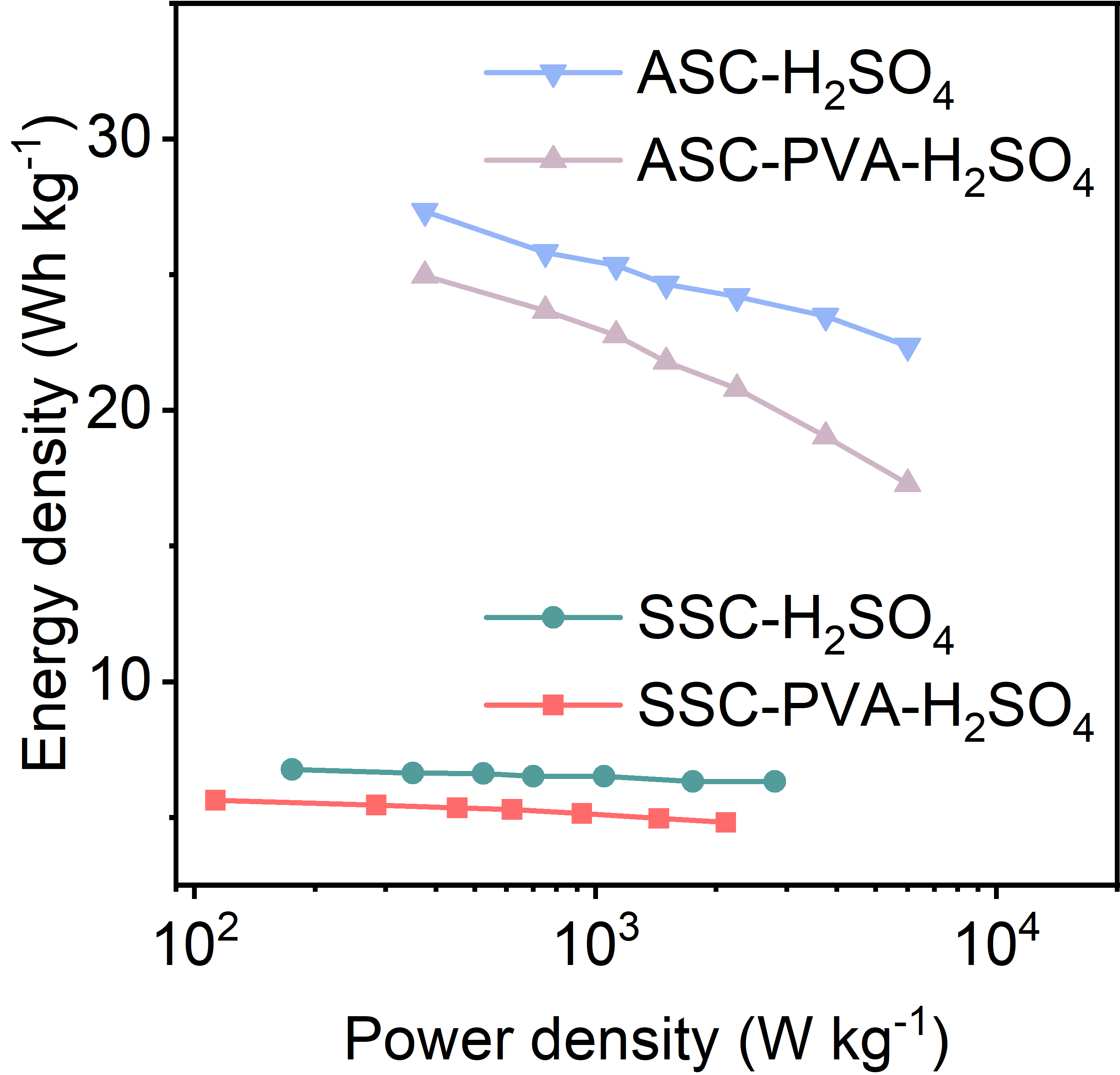
**

**Supplementary Figure 14.** Comparison of power density and energy density relationship between ASCs and SSCs.
